# Comparison of supercapsular percutaneously assisted approach total hip versus conventional posterior approach for total hip arthroplasty: a prospective, randomized controlled trial

**DOI:** 10.1186/s13018-017-0636-6

**Published:** 2017-09-25

**Authors:** Jun Xie, Hongxi Zhang, Lei Wang, Xiang Yao, Zhanpeng Pan, Qinyi Jiang

**Affiliations:** Department of Orthopedics, The Affiliated People’s Hospital, Jiangsu University, Dianli Road 8, Jiangsu Province, Zhenjiang, 212001 China

**Keywords:** Total hip arthroplasty (THA), Minimally invasive surgery (MIS), SuperPath posterior approach, Length of stay (LOS), Harris Hip Score(HHS), Visual analog scale (VAS)

## Abstract

**Background:**

Total hip arthroplasty (THA) has been one of the most successful orthopedic procedures over the past 30 years. Nowadays, the techniques of exposure for THA have undergone great changes, allowing surgeons to perform THA through mini-incisions. Recently, a novel minimally invasive surgical technique of the supercapsular percutaneously assisted total hip arthroplasty was reported in 2011. The purpose of this study was to compare the SuperPath approach with the conventional posterior approach, in terms early outcomes and radiologic results.

**Methods:**

Ninety-two consecutive unilateral primary hip osteoarthritis adult patients were randomly divided into two groups. Forty-six patients (SuperPath group) were operated on using the SuperPath approach, and 46 patients (conventional group) were operated on with the conventional posterior approach. Outcomes were evaluated using preoperative index, intraoperative data, and postoperative function data. The positioning of the implants was analyzed by radiography.

**Results:**

No significant difference was detected in skin-to-skin operation time, blood loss, transfusion rate, postoperative complications, abduction angle, anteversion angle, and stem alignments. The incision length and length of stay (LOS) in the SuperPath group were significantly lower. The VAS score in the SuperPath group at the 1-week, 1-month and 3-month postoperative intervals were lower than those VAS scores in the conventional group. The Harris Hip Score and Barthel Index (BI) for Activities of Daily Living in the SuperPath group were significantly higher at the 1- and 3-month follow-up intervals and were not significantly different 1 year after operation.

**Conclusions:**

This prospective randomized study reveals that the SuperPath technique was associated with shorter LOS, earlier time to walk and climb, and lower postoperative pain levels. It also allowed early postoperative rehabilitation and faster recovery than conventional technique.

## Background

Total hip arthroplasty (THA) has been regarded as one of the most successful orthopedic reconstructive procedures for improving life quality in patients suffering from both trauma and end-stage degenerative joint disease. However, these conventional surgical approaches for THA have their respective shortcomings [1], including longer incisions, massive tissue damage, increased perioperative blood loss, and delayed postoperative rehabilitation. Recently, with the development of minimally invasive surgery (MIS), total hip arthroplasty has undergone an exciting evolution; several mini-incision approaches for THA have been described (Table 1). Although the success of these MIS techniques is well documented [2], there are still some major concerns, such as sheer learning curves, and also suboptimal bone preparation and component malposition due to a limited visualization of the surgical field [3].

**Table 1:** Different minimally invasive approaches for THA

Mini-approaches	Surgical summary
DAA (direct anterior approach)	8–10-cm incision; no cut to the muscles and tendons; anterior capsule removed; need special table or apparatus; femoral implant limited due to poor exposure
Direct lateral	8–10-cm incision; cut gluteus medius and gluteus minimus; limp in some cases postoperation
Posterolateral	6–8-cm incision; split gluteus medius; limited exposure limp in some cases postoperation
Two incisions	Acetabular component placement through an anterior incision and femoral component through an a small posterior incision; longer operation time; sheer learn curve; procedure complexity

In an attempt to overcome these disadvantages, a novel MIS technique and initial experience of the supercapsular percutaneously assisted total hip arthroplasty (SuperPath®, MicroPort Orthopedics Inc., Arlington, TN, USA) was reported by Dr. James Chow in 2011 [4]. This technique was created by combining the percutaneous preparation of the acetabulum through a portal of the PATH approach and the femoral reaming and broaching of the SuperCap approach [5]. This surgical technique does not require any special operative tables or the forced dislocation of the femoral head. The approach utilizes the tissue space between the gluteus medius and the piriformis to access the capsule without releasing the conjoint tendons or external rotator muscles [4]. In relative publications, observations of this surgical technique have shown a low complication rate, satisfactory radiographic outcomes, shortened length of hospital stay, and excellent early functional results [6, 7].

In 2014, the supercapsular percutaneously assisted total hip arthroplasty (SuperPath) was introduced to China. Our hospital is one of the earliest institutions to carry out this surgical technique. In order to conduct further study about SuperPath, the randomized design of this controlled study was used to answer four major questions:

1. Is there less blood loss and transfusion rate in the SuperPath group?
2. Does SuperPath lead to a faster rehabilitation than the conventional group?
3. Does the SuperPath group have better functional outcomes?
4. Does poor exposure in the SuperPath group interfere with the correct position of the prosthesis?

Analysis of the perioperative outcomes, pain relief, and function results was performed, and comparisons between the SuperPath technique and the conventional posterior surgical technique were noted. The traditional posterior technique was selected as a comparator, because it is the most commonly used approach for THA.

## Methods

### Study design

This was a prospective randomized controlled trial of patients with unilateral primary hip osteoarthritis. The study was conducted according to the “CONSORT statement” guidelines for randomized controlled trials [8]. Our trial follows strictly the guidelines of the ethical censorship of Jiangsu University and has been also approved by the Ethics Committee of Zhenjiang First People’s Hospital Affiliated to Jiangsu University (approval number 2014-JSU-EC-041).

### Patients

**Fig. 1:**
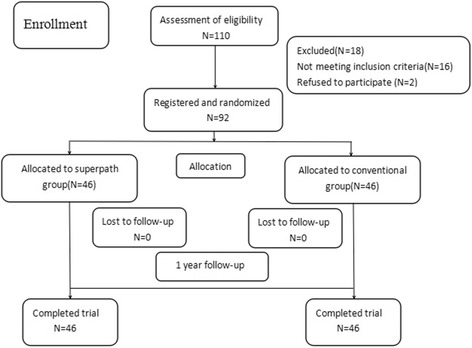
Flow of patients through the study

Between November 01, 2015, and February 28, 2016, a total of 92 consecutive adult patients, who suffered from unilateral primary hip osteoarthritis, were recruited and randomly assigned to two groups who were treated at The Affiliated People’s Hospital of Jiangsu University, China. All patients received the type of treatment to which they had been allocated (Fig. 1), and they were assigned to two groups: group 1: 46 patients were assigned to the SuperPath group and were operated on using the SuperPath approach and group 2: 46 patients were assigned to the conventional group and were operated on using the conventional posterior approach. The patients’ demographics are shown in Table 2. All surgeries were performed by one senior orthopedic chief surgeon. Patients in both groups were implanted with the same cementless THA implants (i.e., acetabular component, acetabular liner, femoral component, femoral head). The diameter of the prosthetic head of 28 mm was used for all implants in both groups. Our exclusion criteria were femoral neck fracture, severe acetabular defect, metastatic disease, and overweight patients with a body mass index over 40. These patients were followed up in the same rehabilitation unit. Functional outcomes were evaluated using the following measures: Harris Hip Score (HHS), Barthel Index (BI), and visual analog scale (VAS) for pain level, TUG (a timed physical examination that evaluates the time it takes a subject to stand from a seated position, walk 3 m, and return to a seated position), TSC (the time needed to go up and down a flight of 12 stairs). Evaluations were conducted at 1-week, 1-month, 3-months, and 1-year post-operation intervals. Other results included incision length, blood loss, skin-to-skin operative time, and also length of stay (LOS), as well as complication rates. The positioning of the implants was analyzed by radiography. Each patient will be informed both orally and in writing with complete details about the procedure, the possible risks, the voluntary nature of the participation, and the right to withdraw at any moment prior to enrollment. Informed consent will be required to be signed by each patient before his/her entry into the trial and will be kept in the research archives.

**Table 2:** Preoperative patients’ demographic characteristics in SuperPath group and conventional group

	SuperPath group	Conventional group	*P* value
No. of patients	46	46	–
Age (years)	66.60 ± 11.88	64.47 ± 12.09	0.51
Gender(F/M)	12/34	19/27	0.12
BMI (kg/m^2^)	23.62 ± 1.63	24.06 ± 2.72	0.31
VAS	7.62 ± 1.63	7.06 ± 1.72	0.53
Harris Hip Score	28.9 ± 11.32	29.3 ± 17.40	0.40
Barthel Index	68.9 ± 8.35	65.3 ± 7.64	0.13

### SuperPath approach technique

**Fig. 2:**
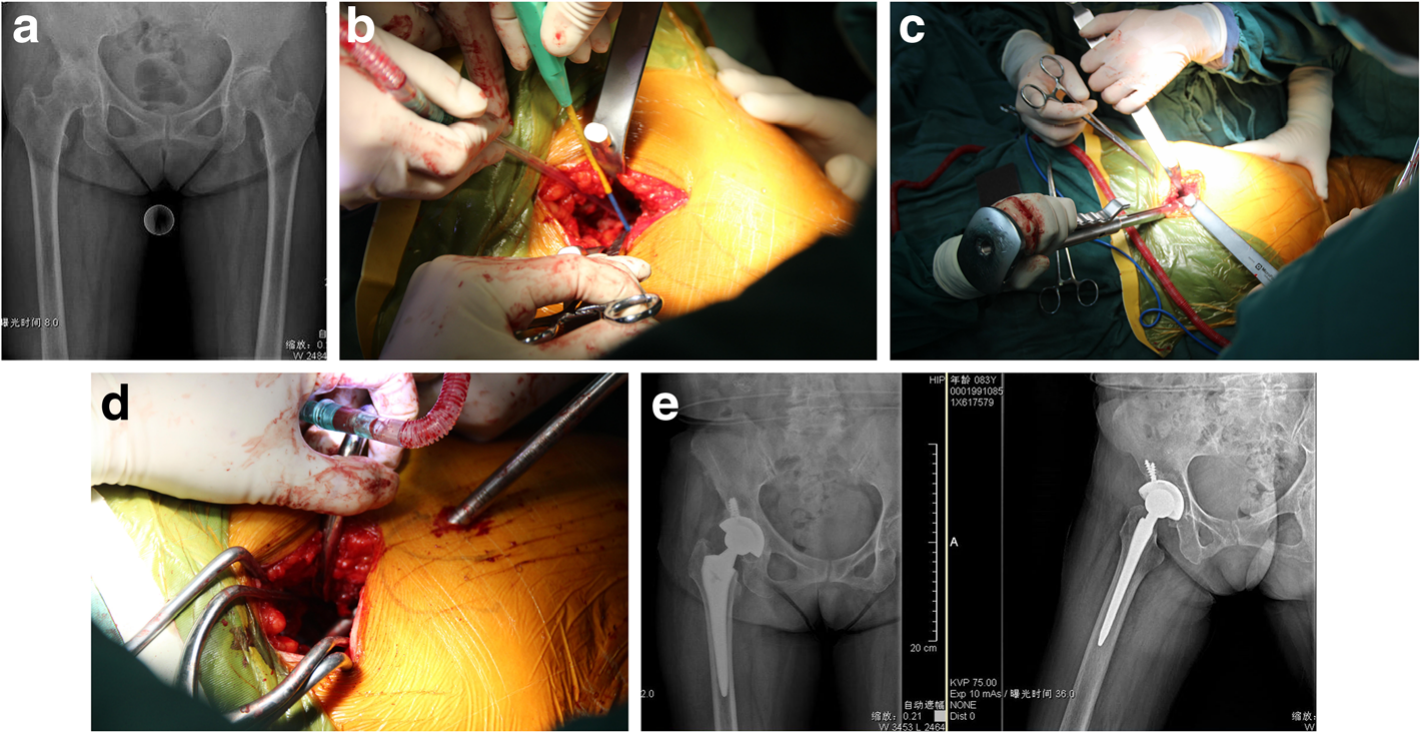
A patient suffered from right hip osteoarthritis (**a**). Following the initial incision, two wing-tipped elevators were used to split the gluteus maximus muscle and expose the underlying gluteus medius muscle (**b**). Sequential femoral broaches were then used to complete preparation and size the proximal femoral canal (**c**); use an appropriately sized acetabular basket reamer to ream the acetabulum through the main incision and connected to the reamer drive shaft inserted through the cannula proximally into the main incision through a 1-cm incision located 1 to 2 cm posterior to the femur (**d**). Postoperative standard anteroposterior and lateral radiographs (**e**)

The patient was positioned in the lateral position with the hip in 45° of flexion and 10–15° of internal rotation. A 6–8-cm incision superior to the greater trochanter was made [6]. The gluteal fascia was incised, and the gluteus maximus was separated in line with fibers. The interval between the gluteus minimus and piriformis was exposed by using a Zelpi retractor. One blunt Hohmann retractor was placed anteriorly under the gluteus medius to protect the muscle, and the leg was elevated to reduce the tension on the external rotators making it easier to place another Hohmann retractor beneath the piriformis to protect the sciatic nerve. A Cobb elevator was used to push the posterior part of the gluteus minimus muscle anteriorly and expose the hip joint capsule. The hip joint capsule was then cut according to the incision from the base of the greater trochanter to 1 cm proximal to the acetabular rim. The capsule was elevated as a flap anterior and posterior to improve visualization, and the blunt Hohmann retractor was then moved to the intracapsular position. Starting in the anterior portion of the piriformis fossa, the femur was reamed and broached without dislocation. Occasionally, in osteoarthritis patients, huge osteophytes need to be removed by osteotome to expose the starting point. An entry reamer was used to open the canal, and a canal feeler was used to confirm the position in the canal. A calcar punch was used to knock out the femoral neck and head in order to insert the broaches. Consecutive broaches were used until the appropriate broach was placed, and depth relative to the greater trochanter was compared to the preoperative plan. The femoral neck osteotomy was made using the superior aspect of the broach as a guide and two Schanz pins were inserted into the femoral head in order to rotate and remove the head. The femur was then displaced anteriorly by the assistant using a bone hook. The implant trial cup was placed into the acetabulum**.** A portal placement guide was used to allow for the placement of a reaming cannula just posterior to the trochanter in line with the planned acetabular placement. The cannula was left in place, and extraction was made using a portal placement guide. The cannula was kept close to the femur to ensure that it was well away from the sciatic nerve. The acetabulum was prepared by resecting calcified labrum and ensuring that the transverse acetabular ligament remained visible. An appropriately sized acetabular basket reamer was inserted in the acetabulum through the main incision and connected to the reamer drive shaft through the cannula, allowing reaming with preservation of the external rotators. The definitive cup and polyethylene liner were placed in a similar procedure (using a portal placement guide) with the option for alignment guides. A trial head and neck were placed, and a blunt trocar was used to push the femur with an assistant adducting the leg and rotating the femur to reduce the neck into the femoral head. C-arm fluoroscopy was used in order to ensure that the trial component position and angulation were correct. Components were then separated and removed. The definitive femoral head was inserted, and a femoral prosthesis was implanted and reduced again. The hip joint capsule was perfectly preserved and closed with a suture. Then, the gluteal fascia and skin were closed with sutures (Fig 2).

### Posterior approach technique (Moore approach)

The patient was placed in a lateral position; the incision was started 10 cm distal to the posterior superior iliac spine and extended to the posterior margin of the greater trochanter. The length of the incision was 12–13 cm; exposure and division of the deep fascia was made in line with the skin incision. The fibers of the gluteus maximus were dissected bluntly and separated, and exposed the greater trochanter. Divisions of the distal fibers were exposed, and the external rotators were released. The muscles were retracted medially, and the capsule was exposed and split distally to the proximal along the line of the femoral neck in order to detach the distal part of the capsule from the femur the rim of the acetabulum. The standard posterior technique was followed in order to perform the femoral neck osteotomy, the hip was dislocated posteriorly, and the prosthesis was implanted.

### Follow-up

All patients were followed up in the same rehabilitation unit in our hospital. The postoperative outcomes were assessed at 1-week, 1-month, 3-month, and 1-year follow-up intervals after the operation, and standard anteroposterior and lateral radiographs were taken for both groups. The cup abduction angle and the anteversion angle were recorded, and the stem alignment was measured between the anatomical axis and the long axis of the femur. If the angle was under 1° valgus or varus, it was considered as good. These outcomes were recorded by an independent investigator who did not participate in the study.

### Statistics

All statistical analyses were performed using SPSS 12.0 (SPSS, Inc., Chicago, IL, USA). The clinical data and radiographic parameters were expressed as mean ± standard deviation. Differences between two groups were detected using Student’s *t* test and *χ* ^2^ test. The Shapiro-Wilk and Levene’s tests showed normal data distribution and variance. *P* value less than 0.05 was considered statistically significant.

The intention-to-treat (ITT) group is defined as the patients who are randomized. The per-protocol (PP) group is defined as the patients who completed the study and do not have major protocol violations. All analyses were based on the ITT group and PP group.

And the result of the ITT analysis will be compared with that of the PP analysis to check whether the results are consistent.

## Results

All patients were assessed according to their ability to walk weight bearing as tolerated on the first postoperative day. It was observed that 100% patients in SuperPath group mobilized without restriction while the conventional group mobilized with hip precautions for 4 weeks. Both groups showed substantial overall improvement in mobility and function as compared with preoperative status. In the 1-year follow-up, no prosthesis was loosened or subsided. Compared with the conventional group, the incision length and length of stay (LOS) of the subjects in the SuperPath group were significantly lower (Table 3). No significant difference was detected in skin-to-skin operation time, blood loss, or transfusion rate (Table 3). TUG, TSC, and VAS for pain-level scores in the SuperPath group at 1-week, 1-month, and 3-month follow-up intervals were significantly lower than recorded TUG, TSC, and VAS scores in the conventional group. The Harris Hip Score and Barthel Index in the SuperPath group were significantly higher at 1-month and 3-month follow-up, but not significantly different at the 1-year follow-up post-operation (Table 4). The cup abduction angle, anteversion angle, and the positioning of the stem were not different in either group. In the stem positioning, no outliers of more than 5° varus or valgus occurred (Table 5). None of the patients had fractures, postoperative infection, nerve damage, or heterotopic ossification. In the conventional group, a 72-year-old man suffered a deep venous thrombosis, one patient in the SuperPath group suffered dislocation after 1 week, and two patients in the conventional group suffered dislocation after 2 weeks. No significant postoperative complications were observed in either of the two groups.

**Table 3:** Perioperative patients’ data

	SuperPath group	Conventional group	*P* value
Operation time (m)	103.6 ± 11.8	106.5 ± 16.5	0.53
Incision length (cm)	7.4 ± 1.06	14.5 ± 2.38	0.000
Blood loss (ml)	303.6 ± 106.3	326.4 ± 127.2	0.11
Transfusion rate	4.3% (2/46)	11% (5/46)	0.24
Length of stay (days)	8.3 ± 3.6	11.4 ± 2.4	0.000

**Table 4:** Comparison of values for postoperative outcomes between SuperPath group and conventional group

	Follow-up time	SuperPath group	Conventional group	*P* value
VAS	1 week	4.86 ± 0.83	6.53 ± 0.52	0.000
	1 month	2.6 ± 0.82	3.4 ± 0.63	0.009
	3 months	1.4 ± 0.63	1.87 ± 0.74	0.048
	1 year	0.87 ± 0.51	0.97 ± 0.35	0.16
TUG(min)	1 week	2.06 ± 1.43	3.2 ± 1.47	0.002
	1 month	1.33 ± 0.36	2.57 ± 0.59	0.016
	3 months	0.92 ± 0.10	1.2 ± 0.23	0.036
	1 year	0.52 ± 0.12	0.58 ± 0.09	0.70
TSC(min)	1 week	5.34 ± 1.85	7.2 ± 2.04	0.000
	1 month	2.56 ± 0.78	3.47 ± 0.83	0.022
	3 months	1.96 ± 0.69	2.21 ± 0.55	0.041
	1 year	1.06 ± 0.13	1.09 ± 0.19	0.55
Harris Hip Score	1 week	73.8 ± 3.89	69 ± 4.81	0.009
	1 month	81.4 ± 3.18	76.8 ± 2.93	0.000
	3 months	87.6 ± 1.76	80.1 ± 4.49	0.000
	1 year	92.3 ± 1.62	91.6 ± 2.41	0.26
Barthel Index	1 week	70.67 ± 9.47	64.46 ± 7.70	0.000
	1 month	79.6 ± 10.01	74.26 ± 5.76	0.017
	3 months	90.26 ± 7.12	83.07 ± 8.62	0.01
	1 year	94.33 ± 6.90	93.60 ± 8.74	0.334

**Table 5:** Radiologic evaluation of the position of the implants

	SuperPath group	Conventional group	*P* value
Cup abduction angle	43.6 ± 6.8	44.5 ± 6.5	0.41
Cup anteversion angle	17.4 ± 1.6	18.5 ± 1.8	0.23
Stem alignment neutral	43	44	0.21
Varus	2	1	0.62
Valgus	1	1	–

## Discussion

Total hip arthroplasty is the most commonly performed adult surgery in the past decades since this technique was first performed 100 years ago. Nevertheless, the renovation of THA has been dynamic, and investigations continue to improve along two main paths, including (1) improvement in the durability of the prosthesis and (2) approach modifications in the operation to accelerate rehabilitation. More recently, minimally invasive techniques have been introduced to orthopedic fields and have received widespread attention. Increased interest is occurring in performing THA through modified smaller approaches less than 10 cm or smaller incisions [9, 10]. Some mini-approaches (MIS) for THA have been described (mini-anterior, mini-lateral, mini-posterolateral, two small incisions) in research publications that have demonstrated that MIS THA would lead to decrease of blood loss, less postoperative pain, early quick rehabilitation, and more cosmetically acceptable surgical scars [11–13].

Although success of these modified mini-approaches has been proven, it is very difficult for a surgeon to perform surgery using a new approach when he is not familiar with the anatomical structure. To orthopedics, most of them were familiar with the posterior approach and this technique may allow good exposure to hip capsule, ease the insertion of implants, and enable maintenance of abductor strength and also lower the blood loss during surgery than other approaches [14, 15]. Even though it generally results in a higher dislocation rate, the traditional posterior approach is still the most commonly used approach for THA [16]. The anatomic groundwork of SuperPath is similar to posterior approach technique, and no special operative tables or equipment, aside from the supplied instrumentation, are required. Therefore, it is easier to perform MIS by using SuperPath compared with other mini-approaches. It can also be easily extended to a familiar posterior approach if and when surgical complications occur.

In our study, compared with the conventional group, observations in the SuperPath indicate many MIS advantages, such as decreased postoperative pain, shorter LOS, accelerated rehabilitation, and better early postoperative function [7, 17]. Our data supported Bodrogi’s conclusion that the SuperPath approach allows for tissue sparing through preservation of external rotators, minimizing stretching of the gluteus medius, and reducing postoperative pain. These improvements likely lead to decreased postoperative narcotics usage and enhanced postoperative abductor strength and fast recovery that may all contribute to shortened hospital stays [18]. Subjects’ hip muscle activity ability was evaluated by TUG and TSC, which showed obvious improvement in walking speed and climbing ability in the SuperPath group, which could be biomechanically related to less impairment of hip muscles [19]. The significance of subjects’ earlier ability to walk alone and to climb stairs unassisted illustrated the increased overall function of this new MIS technique with respect to the postoperative rehabilitation. As a result, the higher Harris Hip Score and Barthel Index were observed in the SuperPath approach compared to the conventional approach.

Another potential benefit to patients undergoing the SuperPath technique is the theoretical decreased risk of posterior dislocation by the reason of intact external rotator muscles and the repaired joint capsule. A recent randomized analysis reported that preservation of the external rotators would reduce dislocation rates from 6.2 to 1.8% [20]. In this study, with the SuperPath approach, the dislocation rate was 2.2% (1/46), and in contrast to the posterior approach, the dislocation rate was 4.3% (2/46). Although no statistical preoperative differences between subjects in the two groups were noted, in fact postoperatively, all subjects in the SuperPath group mobilized without restriction, while in conventional group, all subjects mobilized with hip precautions for 4 weeks. Therefore, we agreed the results using the SuperPath technique would reduce the dislocation rates.

In the experience of other authors [21], the SuperPath technique decreased blood loss and transfusion requirements as compared to the conventional posterior approach, but we did not detect the same results. Both groups experienced small amounts of bleeding in operation, because bleeding of THA is mainly due to the bleeding of osteotomy surfaces and in the medullary cavity. Although some investigators attribute much bleeding to dissect piriformis, obturator, gemellus superior, and gemellus inferior, they thought preservation of these muscles in MIS would reduce bleeding significantly. However, according to our experience, using electrocautery to release external rotators close to bone surface slowly will not cause a large amount blood loss in the conventional posterior approach.

One considerable controversy in the SuperPath technique was implant malposition because of poor exposure. With our radiographic follow-up, there was no difference between two groups on cup abduction angle, anteversion angle, and stem alignment. Traditionally, the Lewinnek safe zone has been shown to be associated with the lowest postoperative dislocation rate which is a cup anteversion of 5° to 25° and abduction angle of 30° to 50°, thus aiming to achieve a combined anteversion of 25° to 45° [22]. These outcomes provided observations contrary to some investigators’ reports that MIS approaches may lead to component positioning negatively. We believe that such consistency in implant positioning may contribute to the SuperPath technique using a lateral position and reaming the femur without dislocation, which allow for precise measurement of the patient’s physical femoral anteversion. A perpendicular to the ischial tuberosity or a perpendicular to the axis of the body is accepted technique to measure version of the cup in operation, and the visible transverse acetabular ligament and native acetabular were also used to align the acetabular components before inserting the implant. To our practice, both preoperative computed tomography imaging and intraoperative AP radiograph were very useful to assess cup anteversion and abduction angle in MIS THR surgery.

Another potential concern of performing this technique was the periprosthetic fracture. Some researchers suggested that this rate may be increased in MIS approaches in both the arthritic [23] and osteoporotic populations [24, 25]. Nonetheless, there was no such complication in either group. Therefore, we do not feel that this risk is any greater with the SuperPath approach compared to other open approaches. The authors experienced one intraoperative periprosthetic great trochanter fracture when performing SuperPath for treatment of a femoral neck fracture. In this case, the incision was extended and it was easy to use a tension band to fix the fracture because of good intraoperative visualization.

Aside from the benefits to the patient, the use of the described surgical technique also provides potential advantages to the surgeon. There are essentially no restrictions on the implant design. The technique utilizes a posterior approach that is familiar to orthopedic surgeons, as James Chow demonstrated that the learning curve would be stabilized by case 40 for the SuperPath technique [26]. We also suggest that the surgeon should have rich experience in THR and choose femoral neck fracture patients in the first cases. After the first 20 or 30 fracture cases are finished, osteoarthritis patients could be performed as the surgeon becomes more skilled.

### Limitations

There are several limitations to our investigation including the small number of cases and the short-term follow-up. A short follow-up time could result in the missing of complications or information for patients. Another limitation was patients cannot be blinded for the approach chosen. These patients may have preconceptions that they will have less pain and a fast recovery and these positive preconceptions could impact short-term rehabilitation.

## Conclusions

In our investigation, the early results of the SuperPath approach demonstrate safety and reliability in the short term. These results showed that this technique could significantly reduce VAS pain level, shorten LOS, and accelerate early rehabilitation in comparison to the conventional posterior approach—especially that SuperPath allowed unrestricted postoperative mobilization. While long-term studies have not been completed, we believe advantages of the SuperPath procedure will be more significant with the development of MIS THA.

## Abbreviations

HHS: Harris Hip Score
LOS: Length of stay
MIS: Minimally invasive surgery
SuperPath: Supercapsular percutaneously assisted total hip
THA: Total hip arthroplasty
TSC: Is the time needed to go up and down a flight of 12 stairs
TUG: Is a timed physical examination that evaluates the time it takes a subject to stand from a seated position, walk 3 m, and return to a seated position
VAS: Visual analog scale
